# The protist *Trichomonas vaginalis *harbors multiple lineages of transcriptionally active *Mutator*-like elements

**DOI:** 10.1186/1471-2164-10-330

**Published:** 2009-07-21

**Authors:** Fabrício R Lopes, Joana C Silva, Marlene Benchimol, Gustavo GL Costa, Gonçalo AG Pereira, Claudia MA Carareto

**Affiliations:** 1 UNESP – São Paulo State University, Department of Biology, 15054-000, São José do Rio Preto, São Paulo, Brazil; 2 Institute for Genome Sciences and Department of Microbiology and Immunology, University of Maryland School of Medicine, Baltimore, Maryland 21201, USA; 3 USU – Santa Ursula University, Institute of Biological and Environmental Sciences, 22231-010, Rio de Janeiro, Brazil; 4 UNICAMP – State University of Campinas, Department of Genetics and Evolution, 13083-970, Campinas, São Paulo, Brazil

## Abstract

**Background:**

For three decades the *Mutator *system was thought to be exclusive of plants, until the first homolog representatives were characterized in fungi and in early-diverging amoebas earlier in this decade.

**Results:**

Here, we describe and characterize four families of *Mutator*-like elements in a new eukaryotic group, the Parabasalids. These ***T****richomonas ****v****aginalis **Mu**tator- **l**ike **e**lements*, or *TvMULEs*, are active in *T. vaginalis *and patchily distributed among 12 trichomonad species and isolates. Despite their relatively distinctive amino acid composition, the inclusion of the repeats *TvMULE1*, *TvMULE2*, *TvMULE3 *and *TvMULE4 *into the *Mutator *superfamily is justified by sequence, structural and phylogenetic analyses. In addition, we identified three new *TvMULE*-related sequences in the genome sequence of *Candida albicans*. While *TvMULE1 *is a member of the *MuDR *clade, predominantly from plants, the other three *TvMULEs*, together with the *C. albicans *elements, represent a new and quite distinct *Mutator *lineage, which we named *TvCaMULEs*. The finding of *TvMULE1 *sequence inserted into other putative repeat suggests the occurrence a novel TE family not yet described.

**Conclusion:**

These findings expand the taxonomic distribution and the range of functional motif of *MULEs *among eukaryotes. The characterization of the dynamics of *TvMULEs *and other transposons in this organism is of particular interest because it is atypical for an asexual species to have such an extreme level of TE activity; this genetic landscape makes an interesting case study for causes and consequences of such activity. Finally, the extreme repetitiveness of the *T. vaginalis *genome and the remarkable degree of sequence identity within its repeat families highlights this species as an ideal system to characterize new transposable elements.

## Background

Transposable elements (TEs) are ubiquitous components of prokaryotic and eukaryotic genomes and, as a consequence of their prevalence, mobility and concomitant mutagenicity [e.g., [1,2]], they can induce profound changes in genome organization and have an important evolutionary impact on expression and function of host genes [3-6]. TEs can lead to genome expansion and contraction [7-9], transduction and amplification of host gene fragments [10,11] and increase the variability of protein repertories [12-20]. Given this enormous potential as a source of genetic novelty, considerable effort has been devoted by the scientific community to the characterization of new TEs in the plethora of new genomes and transcriptomes available in public databases, particularly in organisms for which the knowledge about TEs is scarce. While some families of TEs are found across most taxa surveyed, others appear to have a restricted host distribution; the *Mutator *system in plants was an example of the latter. This notion was recently dispelled by the identification and extensive characterization of *Mutator *homologs in the first non-plant species [21-24]. Moreover, consensus sequences of new representatives of this TE family obtained from a broad range of species have been reported in Repbase Reports within the past few years: *CEMUDR1-2 *from *Caenoharbidtis elegans *[25,26]; *MuDR1-2_TP *in the diatom *Thalassiosira pseudonana *[27,28]; *MuDr1-2_NV *in the starlet sea anemone *Nematostella vectensis *[29,30]; *MuDR1x-2x_SM *in the planarian *Schmidtea mediterranea *[31,32] and *MuDr1x-2x_AP *in the insect *Acyrthosiphon pisum *[33,34].

The *Mutator *(Mu) system was originally identified by Robertson [35] in maize as a highly mutagenic transposon system. This system is composed of diverse families that share ~220 bp terminal inverted repeats (TIRs) and create a 9 bp host sequence duplication at the insertion site [reviewed by [36]]. These elements can be either autonomous (*MuDR*) or nonautonomous (*Mu*). Transposition of *Mu *elements is dependent of the autonomous *MuDR *elements. The *MuDR *element in maize is 4.9 kb long and contains two open reading frames (ORFs): *mudrA *and *mudrB*. The *mudrA *gene product, the MURA protein of 823 amino acids, probably a transposase, contains a catalytic domain with a D34E motif (aspartic and glutamic acids separated by 34 residues) and its expression is sufficient for the somatic excision of the TE [37,38]. The transposase encoded by *mudrA *shares weak but significant similarity to those encoded by the *IS256 *group of prokaryotic insertion sequences [21]. Deletions on *mudrA *disable the *Mutator *transpositional activity [37]. The MURB protein is encoded by *mudrB*; while this protein's function remains undetermined, it seems to be necessary for the activity of the *Mu *system in maize [37,38]. *Mutator*-like elements (MULEs) have been identified in a wide range of plant species, such as *Arabidopsis *[39-41], *Oryza *[e.g., [42,43]], *Saccharum *[44,45] and different grasses [46]. Interestingly, MULEs lack the *mudrB *gene [36]. In maize, thale cress and rice MULEs are heterogeneous in sequence, size and structure. In particular, some elements either carry small imperfect TIRs or completely lack them [39,40].

Recently, non-plant species have been reported to harbor MULEs. Chalvet et al. [22] provided the first evidence for the presence of an active MULE in the fungus *Fusarium oxysporum*, the transposon *Hop*. It is 3,299 bp long, has TIRs of 99 bp and 9 bp target site duplication (TSD), encodes a putative transposase of 836 amino acids and has no apparent sequence specificity at the insertion site. The presence of related elements in other filamentous fungi like *Magnaporthe grisae*, *Neurospora crassa *and *Aspergillus fumigatus *has also been reported [22]. Neuvéglise et al. [23] identified a new type of DNA transposons, *Mutyl*, in the yeast *Yarrowia lipolytica *with 7,413 bp, imperfect TIRs of 22 bp, 9 to 10 bp TSD, and two ORFs which potentially encode proteins of 459 and 1,178 amino acids. Whereas the first ORF shows no significant homology to described proteins, the second one shows similarity to a wide variety of *MULE*-encoded transposases. More recently, Pritham et al. [24] characterized a canonical copy of the *Mutator*-like element in a protist genome, *Entamoeba invadens*. This element, named *EMULE-Ei1*, is 2,882 bp long and displays structural features typical of plant MULEs, such as TIRs of 187 bp and a 9 bp flanking TSD. Moreover, it contains a single ORF that putatively encodes a 456-aa protein that shows significant similarity to the *Hop *transposase from *F. oxysporum*. In that study, homologous elements were observed in three additional *Entamoeba *genomes, namely *E. dispar*, *E. hystolitica *and *E. moshkovskii *[24].

*Trichomonas vaginalis*, an asexual flagellated protist [47], is an extracellular obligate human parasite of the urogenital tract [48] and a member of a deep-branching eukaryotic lineage, the Parabasalids [49]. Its genome sequence and annotation, published in 2007 by Carlton and collaborators, revealed a putative set of ~60,000, mostly intronless, protein-coding genes, endowing *T. vaginalis *with one of the largest gene sets among eukaryotes [9]. Interestingly, this genome was shown to be highly repetitive, with repeats and TEs comprising about two-thirds of its ~160 Mb-long sequence. Until now, only DNA transposons have been completely characterized in this species, including *Mariner *[50], *Polintons *[51], and *Mavericks *[52]. Among the original repeats identified in the genome of *T. vaginalis *were included four repeat consensus sequences with a *Mutator*-like profile: R210 with 2,127 bp, R130a with 1,129 bp, R119 with 2,954 bp, R165 with 2,410 bp [9]. In this report, we characterize these four *T. vaginalis Mutator*-like elements (*TvMULEs*), which we renamed as *TvMULE1 *(based on the R210 sequence), *TvMULE2 *(based on the R130a sequence, here revised regarding to sequence and structure), *TvMULE3 *(based on R119) and *TvMULE4 *(based on R165). We confirm the inclusion of the four repeats into the *Mutator *superfamily based on sequence, structural and phylogenetic analyses. While *TvMULE1 *is a member of the *MuDR *clade predominantly from plants, the other three *TvMULEs *represent a new and quite distinct *Mutator *lineage, expanding the taxonomic distribution and the range of functional motif of *MULEs *among eukaryotes.

## Results

### Characterization of *TvMULEs*: new *T. vaginalis *transposons

**Figure 1:**
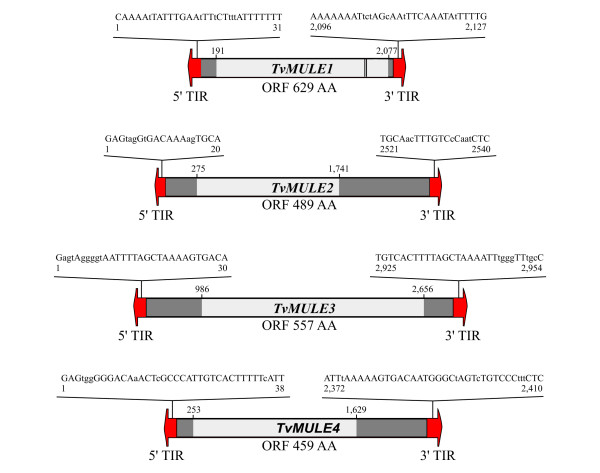
**Structure of the *T. vaginalis MULEs***. Putative terminal inverted repeats (TIRs) are denoted by black arrowheads at each end of the elements. Bases that are variable between TIRs are in lowercase type. Dark gray boxes represent internal non-coding sequences. The internal region of each element (clear gray box) corresponds to an ORF that encodes putative *MULEs*-related transposase domains. Location of a transposase zinc finger (double black lines) is also shown.

The sequence and structure of four *Mutator*-like consensus sequences [9] were analyzed in detail in the present study. The manual inspection of a combination of sequence similarity searches and consensus sequence building techniques (described in Methods) and the presence of putative, imperfect, terminal inverted repeats (TIRs) resulted in the definition four new *Mutator*-like transposable element families represented by the consensus sequence of which we termed *TvMULE1*, *TvMULE3 *and *TvMULE4 *(Figure 1) and *TvMULE2 *represented by the canonical copy contained in the contig 95978 (position 24930–27469).

All insertions of the four families were identified in the 17,290 contigs that make up the current genome assembly of *T. vaginalis *by BLASTN. A total of 61, 514, 666 and 1,204 matches revealed strong similarity to *TvMULE1, TvMULE2, TvMULE3 *and *TvMULE4*, respectively (identity >80% and E ≤ e-20). All matches were extracted by BLAST coordinates and all ORFs starting at the Met residue were predicted, excepting *TvMULE2*, in which the predicted ORF was only derived of the canonical copy. The four *TvMULEs *contain a single intronless gene. The more frequent ORF of *TvMULE1*, which putatively encodes a 629-aa protein (Figure 1), displayed highest similarity (43%) to a *Mutator *transposase (Tpase) from *A. thaliana *(Table 1). On the other hand, the other three *TvMULEs *showed similarity to three potential *Mutator *Tpases from the pathogenic yeast *Candida albicans*: the first of these (GenBank gi # 68466572) is 568-aa residues long and is very similar to the second *C. albicans *protein (GenBank gi # 68466277), which is 832-aa long; the third *C. albicans *protein (GenBank gi # 68474652), is 668-aa long. *TvMULE2 *matched the first *C. albicans *protein, and *TvMULE3 *and *TvMULE4 *showed significant similarity to the third protein with 40 and 43% similarity, respectively (Table 1). While *TvMULE1 *have relatively small non-coding regions, these extend to several hundred base pairs in *TvMULE2, TvMULE3 *and *TvMULE4*.

**Table 1:** Characteristics of 4 *Mutator-*like families in the *T. vaginalis *genome

**Family**	**Lenght^a^** **(bp)**	**TIRsb** **(bp)**	**ORFc** **(aa)**	**First TE hit in BlastP searches against *Genbank***				
				**Description^d^**	***e*** **value**	**%** **ID^e^**	**%** **Similarity^f^**	**Length^g^** **(aa)**
*TvMULE1*	2,127	31	629	11994228 *Arabidopsis thaliana Mutator*	1e-09	27	43	283
*TvMULE2*	2,540	20	489	68466572 *Candida albicans Mutator*	3e-03	24	44	166
*TvMULE3*	2,954	30	557	68474652 *Candida albicans Mutator*	3e-09	23	40	309
*TvMULE4*	2,410	38	459	68474652 *Candida albicans Mutator*	9e-12	25	43	235

^a ^Length of consensus sequence, excepting *TvMULE2 *in which a canonical copy was characterized;

^b ^Putative imperfect terminal inverted repeats;

^c ^Length of the protein encoded by *T. vaginalis *TE, in amino acids (aa);

^d ^GenBank accession number, species, TE name;

^e ^Percent identity between *T. vaginalis *TE-encoded protein and hit in BlastP alignment;

^f ^Percent similarity between *T. vaginalis *TE-encoded protein and hit in BlastP alignment;

^g ^Length of *query *in the alignment produced by BlastP, in amino acids.

Within each of the four *TvMULE *families all copies were found to be nearly identical in sequence (identity >99%). This result confirms the low polymorphism obtained from average pairwise differences between copies (π) observed by Carlton et al. [9]. There, the π value was estimated as 0.9% for *TvMULE1*, 0.7% for *TvMULE2*, 1.1% for both *TvMULE3 *and *TvMULE4*. Within each family, the sequences of the 5' and 3' TIRs are nearly identical. In addition, an alignment of these putative TIRs across *TvMULE *families shows three positions in the 5' end and six in the 3' end are nearly perfectly conserved (not shown). The presence of polymorphism in the terminal ends within each repeat family could indicate that they do not act as the transposase recognition site, given that the internal regions of different copies are more highly conserved. Alternatively, it is possible that the binding is not specific across the entire TIR, or that some of the mutations that have accumulated since transposition actually inactivates the respective copies.

**Figure 2:**
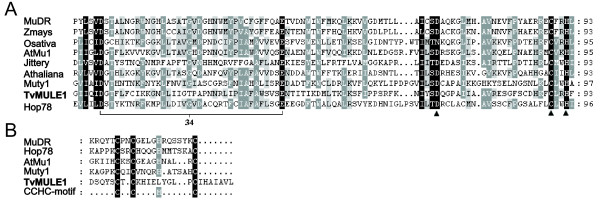
**Conserved domains in the *Mutator *protein MURA and its homologs are present in *TvMULE1***. A – Multiple sequence alignment of the conserved transposase domain. This alignment includes the MURA transposase from the *Zea mays MuDR *element (accession no. 540581), putative MURA-related transposases from the plants *Zea mays *(Zm-40034: accession no. 23928448, *Jittery*: accession no.7673677), *Arabidopsis thaliana *(*AtMu1*: accession no. AC002983.1 and At-96881: accession no. 34914922), *Oryza sativa *(Os-918808: accession no. 8777291), from the fungi *Fusarium oxysporum *(*Hop-78*: accession no. 30421204) and *Yarrowia lipolytica *(*Mutyl*: accession no. 50556866), and from the unicellular protozoan *Trichomonas vaginalis *(*TvMULE1: *deposited in Repbase). B – Multiple sequence alignment of the zinc finger domain. Identical amino acids are shaded in black, and similar amino acids are shaded in gray. The well-conserved D34E integrase signature in the active site of *Mutator *is noted. The symbol (*dark filled triangles*) below of the alignment corresponds to other residues also well conserved across a wide range of *Mutator*-like elements, previously described by Lisch [36].

*TvMULE1 *shares recognized *MULE *structural motifs. Firstly, it has a well-conserved D34E integrase signature in the putative active site, and three residues of the transposase core conserved across a wide range of *MULEs *[36] are also present (Figure 2A). This conserved region corresponds to the ~130-aa domain identified by Eisen et al. [21] containing a 25-aa signature sequence [D-x(3)-G-(LIVMF)-x-(6)-(STAV)-(LIVMFFYW)-(PT)-x-(STAV)-x-(2)-(QR)-x-C-x(2)-H]. Secondly, a transposase zinc finger domain at the C-terminal region was identified, which has a nearly perfect CX_2_CX_4_HX_4/6_C-motif (Figure 1 and Figure 2B). This motif is found in the nucleocapsid protein of retroviruses, in several known nucleic acid binding proteins, in the *copia*-like retrotransposons from tobacco [53], and in *Ty *elements in yeast [54]. It has been proposed that this motif plays a role in a transposase-transposon interaction that takes place during transposition and/or regulation [40].

**Figure 3:**
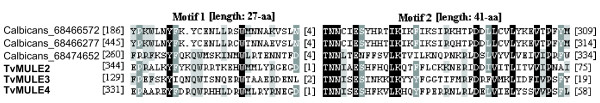
**Clustal alignment of two conserved motifs found in *TvMULEs *and in *C. albicans *homologous sequences**. The number of amino acid residues omitted, which flank and separate the motifs, is indicated in brackets. Residues with related physical or chemical properties are shaded in black when present in all sequences and in gray if present in four out of six sequences.

The other three *TvMULEs *(*TvMULE2*, *TvMULE3 *and *TvMULE4*) show amino acid residue contents that differ markedly from that of *TvMULE1 *and from those of known plant *MULEs*. However, these elements exhibit significant similarity to three *C. albicans *elements (Table 1). This observation is readily apparent from the quite new and distinct content of residues contained in two conserved motifs shared by these six elements (Figure 3). The inclusion of this extended group in the *Mutator *superfamily is supported by a variety of structural analyses. First, the three *C. albicans *proteins show significant similarity to *MULEs *such as *Hop *from *F. oxysporum *(GenBank gi # 30421204) and a *Cucumis melo MULE *(GenBank gi # 46398239); in addition, one of them (GenBank gi # 68466572) contains a conserved *Mutator*-like transposase domain corresponding to pfam00872 (COG3328 and CDD85084), a hallmark of Tpases of the *Mutator *family. Secondly, BLASTP generated significant pairwise alignments for all comparisons between these *TvMULEs *(2e-37<E-value<2e-13), as well as between them and the *C. albicans *sequences (Table 1). Thirdly, a careful characterization of motifs across 41 *Mutator *elements, as well as in these *T. vaginalis *and *C. albicans *repeats, revealed that the latter encode an extended motif of 36 residues (*motif 1*) identical to the 25-aa signature sequence of the *MULE *transposase core previously mentioned [see Additional file 1]. The high degree of sequence conservation of this motif [see Additional file 1] in quite distinct branches of the *Mutator *lineage suggests that it plays a role that is essential to the fitness of the elements.

Finally, none of the four *TvMULEs *encodes a *mudrB *product, similarly to what is observed in the *A. thaliana *and *O. sativa *[40,41,43]. Even in plants, while *mudrA *sequences are widespread in grasses, *mudrB *sequences seem to be restricted to *Zea *[46].

### Preferential insertion sites of *TvMULEs*

Among all matches with similarity to *TvMULE1 *(61) and *TvMULE2 *(514), only 8% (five sequences) and 0.5% (three sequences), respectively, correspond to complete copies. Probably due to their longer size, which can not be spanned by two PCR reads, matches to *TvMULE3 *(666) and to *TvMULE4 *(1,204) represent only internal or end regions of the elements; these observations reflect the fragmentary nature of the current assembly, which in turn is caused by the highly repetitive character of the *T. vaginalis *genome. Thus, the analyses of putative insertion site preferences were performed with all insertions that contain at least one end region.

The sequences flanking *TvMULE1 *insertions exhibit a high degree of nucleotide conservation in the first 25 positions (data not shown). Genomic fragments of 2,000 or 5,000-nt adjacent to the element were extracted to evaluate the extent of such similarity in the regions flanking of different copies. The extent of the similarity between regions flanking *TvMULE1 *insertions depends on the copies of this family being compared. Interestingly, one pair of *TvMULE1 *copies (contig 85938:11024–17138 and contig 91860:9141–15539) appears to be nested within another repeat. In fact, the similarity upstream and downstream of these copies extends to 1,246-bp and to 3,075-bp, respectively, including putative 36-bp TIRs (5'-GgGtcaTTATtGATTTTGTAATTTAATCGTcgTCGT-3', and 5'-ACGAtaATGATTAAATTACAAAATCgATAAcctCtC-3'), suggesting an unknown repeat of approximately 4,300 bp in length. This unknown repeat is itself flanked by two different TSDs (Table 2). Despite the fact that this full-length nested configuration is observed only in the two genomic regions mentioned above, multiple partial copies of *TvMULE1 *that contain one end region are flanked by fragments of this unknown repeat. Sequence similarity searches of this novel repeat against consensus sequences of *Trichomonas *and *Entamoeba *genera stored in Repbase database, ~55 repeat families identified in the *T. vaginalis *genome [9] and Genbank showed no significant matches. Therefore this element remains unidentified. We hypothesize that a copy of this repeat containing an insertion of *TvMULE1 *has transposed in a recent past producing multiple nested copies. However, detailed empirical studies of excision/transposition/insertion by transfection in new lineages are required to corroborate this hypothesis.

**Table 2:** Putative TSDs flanking *TvMULEs *and the *unknown *repeat

**Family**	**TSD**	
	
	**Length (bp)**	**Sequence**
***TvMULE2***	**10**	ATATATCGGC
		TTTATCGCTG^a^
	**11**	AATTGATGAAA
		CCTTAATTCAA
		CCATTTTGATA
		TAATTCTCCAT
		TTTCCCTGAAA
		TGGTTTTATGA
		GAAACAATTAA
	**12**	TTAAATTACTTC
	**14**	AATTAAAAAAATAT
***TvMULE3***	**11**	CTATTTAAAAG
		TTTTTTGATAA
		TTTAAGGTGTT
***TvMULE4***	**12**	AAAAAATTTTGA
		AATTTTTTCGAA
		ATATATCTTTAA
		ATTTTTGAAAAA
		TATACATATATA
		TTATTATTTTAA
		TTTCTTTTTTAT
	**13**	AAAAATTTTGAAA
		ATTTTTTCTGGAT
		AGATTTTTGAAAA
		CTTATTTTTTGAA
		TTTCAAAATTTTT
***Unknown***	**8**	TAGATTTT^b^
	**9**	ATCAAAAAG^c^

^a^Duplication upon the chosen canonical copy;

Duplication upon the insertion contained in the: ^b^contig 85938 and ^c^contig91860.

*TvMULE2*, *TvMULE3 *and *TvMULE4 *are flanked by completely variable regions upstream and downstream of all insertions (data not shown). Curiously, multiple TSDs with distinct lengths are observed, a characteristic not found in *MULEs *previously characterized (Table 2). Taken at face value this would suggest an extreme flexibility in their insertion sites.

Finally, as the genomic distribution of these repeats is putatively the product of only self-mobilization, we assessed the preferential insertion of these *TvMULEs *relative to local GC content calculated in the first 100, 2,000 and 5,000-nt. The average GC content within the nearest 100-nt is 26.9% (se = 0.0) for *TvMULE2*, 27.7% (se = 0.4) for *TvMULE3 *and 25.0% (se = 0.3) for *TvMULE4*. The average GC content in the 2,000-nt and 5,000-nt flanking regions is slightly higher, ranging between 31.3% and 31.8% ± 0.0 for *TvMULE2*, 30.9% and 31.6% ± 0.2% for *TvMULE3*, 30.0% and 30.7% ± 0.2% for *TvMULE4*, respectively. This nucleotide composition is similar to that of intergenic regions in the current assembly (28.8%) and considerably lower than the GC content of *T. vaginalis *genes (53.5%), suggesting either that these two *TvMULE *families insert preferentially in non-active regions or that insertions into genes have been eliminated by selection. This is not unexpected since almost all *T. vaginalis *genes are intronless and TE insertions within coding regions are frequently associated to deleterious effects [e.g., [55]].

### Phylogenetic relationship of *TvMULEs*

**Figure 4:**
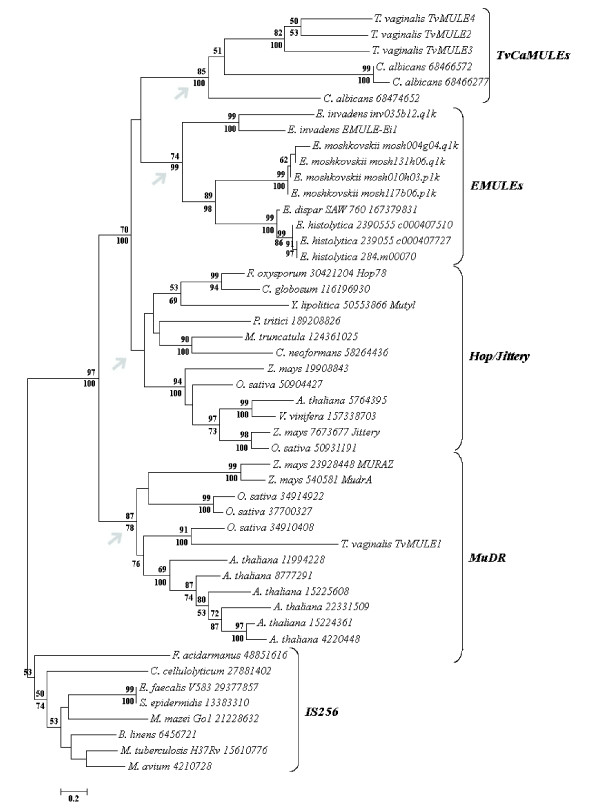
**Phylogenetic tree of *Mutator *superfamily proteins**. The cladogram was generated by neighbor-joining, from an alignment of three conserved amino acid motifs present in all sequences (length = 123 residues), and which corresponds to pfam00872 (COG3328 and CDD85084). The sequences are identified by the host names, GenInfo Identifier (gi) and TE names, when previously characterized. Node support obtained from 1,000 bootstrap replicates using NJ and from their representation in the posterior sample of the bayesian analysis is shown above and below the branches, respectively. Gray arrows indicate the four main clades in the *Mutator *phylogeny.

Three major clades of eukaryotic elements have been identified to date in the *Mutator *superfamily: (1) the *MuDR *group, characteristic of plant genomes, contains the original *Mutator *elements identified in maize, and its relatives from *Arabidopsis *and rice, (2) the *Hop/Jittery *group contains elements from a variety of host taxa including plants and fungi, and (3) the *EMULE *clade, which contains all elements identified in the genome of *Entamoeba *species. Members of these three clades were used to determine the phylogenetic placement of the *T. vaginalis MULE*s in the *Mutator *superfamily, and the tree was rooted with elements belonging to the *IS256 *clade of bacterial transposons. Bayesian analyses showed strong support for the monophyly of the eukaryotic *Mutator *sequences relative to the bacterial *IS256 *elements (Figure 4). The eukaryotic clade is present in 100% of the trees in the posterior sample, a result that is confirmed by neighbor-joining (NJ) analysis (97% bootstrap support). There is also strong support (87% in NJ bootstrap and 78% in bayesian analysis) for a clade containing the *MuDR *elements. The NJ analysis suggests the monophyly of the *Hop/Jittery *clade but the support from Bayesian and NJ bootstrap analyses is <50%. Finally, the *EMULE *sequences form a strongly supported monophyletic clade (74% in NJ bootstrap and 99% in bayesian analysis). The elements from *T. vaginalis *are nested within the broad clade of eukaryotic *Mutator *elements. *TvMULE1 *clusters with an element from *O. sativa *in the *MuDR *clade. On the other hand, *TvMULE2*, *TvMULE3 *and *TvMULE4*, together with the *C. albicans *sequences, form a monophyletic clade present in 100% of the trees in the posterior sample of the bayesian analysis and in 85% neighbor-joining bootstrap trees. All these findings lead us to conclude that the *TvMULEs*/*C. albicans *clade represents a new and quite distinct branch in the *Mutator *superfamily, which we name *TvCaMULEs*.

The genetic distances within and between clades were calculated in order to determine the heterogeneity of the *MULEs*. First, the *TvCaMULE *members are more divergent regarding on the number of amino acid substitution per site (aa/site) among each other (aa/site= 1.84 ± 0.17) than the members of other clades (*Hop/Jittery*: aa/site= 1.53 ± 0.06; *MuDR*: aa/site= 1.45 ± 0.07; *IS256*: aa/site= 1.27 ± 0.08; and *EMULEs*: aa/site= 1.04 ± 0.09). However, this higher divergence is due to difference between the members of the two species (aa/site= 2.19 ± 0.07) than between *C. albicans *(aa/site= 1.43 ± 0.7) and the *TvMULEs *(aa/site= 1.2 ± 0.1) sequences. Second, a pairwise comparison between clades shows that *TvCaMULEs *are the most distinct from any other clade (aa/site= 2.96 ± 0.15) than all other comparison pairs (aa/site= 2.5 ± 0.06). These data lead us to conclude that *TvCaMULEs *form a heterogeneous group and that they are distantly related to the other *MULEs *analyzed.

### Multiple conserved motifs in *Mutator* and *IS256* superfamilies

Forty eight *Mutator and IS256 *transposon sequences were used to search for sequence motifs common within this superfamily. Twelve conserved motifs were identified, with motifs 1, 4 and 8 present in all sequences [see Additional file 2 and Table 3]. Interestingly, only the elements of the *MuDR *and *Hop/Jittery *clades present the D34E active site integrase signature between motifs 4 and 8, while the bacterial transposons show a range in the number of intervening residues in this region (D38-40E) [Table 3]. Some motifs are clade-specific, such as motifs 5 and 9 in the *IS256 *clade, which are similar to the *Mutator*-like transposase domain, while others are more widespread, such as motifs 7 and 10 harbored by plants and fungi in the *MuDR *and *Hop/Jittery *clades.

**Table 3:** Characterization of 48 *MULEs *analyzed in this study

**Clades**	**Analyzed sequences**	**Conserved motifs**		
		
		**All clades**	**Dispersed**	**Clade-specific**
***IS256***	8	1^a^, 4 and 8^c^	-	5^d ^and 9^d^
***MuDR***	12	1^a^, 4 and 8^b^	7^e ^and 10^f^	-
***Hop/Jittery***	12	1^a^, 4 and 8^b^	7^e ^and 10^f^	11^f^
***EMULEs***	10	1^a^, 4 and 8	-	2^f^, 3^f ^and 6^f^
***TvCaMULEs***	6	1^a^, 4 and 8	10^f^	12^f^

**TOTAL**	**48**	**12**		

^a ^25-aa signature sequence described by Eisen et al. [19];

Active site residues found into motifs 4 and 8: ^b^D34E and ^c^D38-40E;

Motif similar to *Mutator*-like transposase domain corresponding to: ^d ^pfam00872 and ^e ^pfam03108;

^f ^No match between motif and putative conserved domains have been detected.

### Distribution and transcriptional activity of *TvMULEs* in Trichomonads

**Figure 5:**
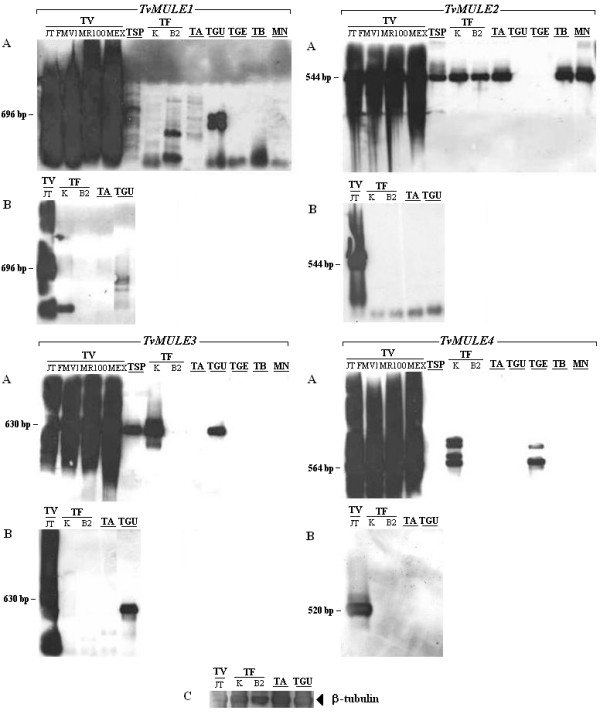
**Detection of *TvMULEs *in trichomonad species by DNA and cDNA hybridizations**. A – Host distribution; B – Transcriptional activity; and C – Hybridization of beta-tubulin controls from each sample to control for RNA loading. TV: *Trichomonas vaginalis *(strains – JT, FMV1, MR100 and Mex); TSP: *Tetratrichomonas sp*; TF: *Tritrichomonas foetus *(strains – K and B2); TA: *Tritrichomonas augusta*; TGU: *Tetratrichomonas gallinarum*; TGE: *Trichomonas gallinae*; TB: *Trichomitus batrachorum*; MN: *Monocercomonas sp*. *Numbers *represent expected size of the amplified fragments.

The low degree of sequence polymorphism within *TvMULE *families suggests a very recent expansion of *Mutator*-like transposons in the *T. vaginalis *genome, either due to TE-induced proliferation or to small-scale duplications of the host genome. To evaluate whether this expansion occurred before or after the global expansion of *T. vaginalis*, four *T. vaginalis *isolates obtained from different geographical regions were analyzed for the presence of *TvMULE *homologs (Table 4). PCR products from each sample were obtained using primer pairs from each canonical MULE family of *T. vaginalis *(Table 5). The specificity of these amplifications was confirmed by stringent DNA hybridizations using as probe an internal fragment of Tpase isolated of the *T. vaginalis *JT strain. The strong hybridization signal in all lanes suggests the presence of all *TvMULEs *in the four *T. vaginalis *strains tested (Figure 5A). Interestingly, homologs to the *TvMULEs *occur in other Trichomonad species, even though their distribution appears to be patchy. All non-*T. vaginalis *isolates showed extremely weak or nearly imperceptible PCR amplification (data not shown), possibly due to low copy number and/or high sequence divergence in the primer region. However, positive hybridization signals were still detected against these amplicons in some of these species (Figure 5A). In particular, *Tetratrichomonas sp *and *T. gallinae*, the two closest species to *T. vaginalis *examined, show evidence of *TvMULE1*, *TvMULE2 *and *TvMULE3*, and of *TvMULE4*, respectively. On the contrary, the species more distantly related to *T. vaginalis *[47] show a heterogeneous pattern. *T. foetus*, a parasite of the urogenital tract in cattle, shows hybridization to each of the four repeats in at least one of the strains sampled, and *T. augusta*, *T. batrachorum*, and *Monocercomonas sp *show evidence of only *TvMULE2*. The patchy distribution among species and strains suggest extensive divergence and/or loss of elements homologous to *TvMULEs *among Trichomonads.

**Table 4:** *Trichomonad *species and strains used in this study

**Species**	**Isolates**	**Origin**	**Host**	**Hybridization**	
				
				**DNA**	**cDNA**
*Trichomonas vaginalis*	JT	Rio de Janeiro/Brazil	Human	✔	✔
	FMV1	Minas Gerais/Brazil	Human	✔	
	MR100	Czec Republic^1^	Human	✔	
	Mex	Mexico	Human	✔	
*Tetratrichomonas sp*	SP1	Argentina	Pigeon	✔	
*Tritrichomonas foetus*	K	Rio de Janeiro/Brazil^2^	Bovine	✔	✔
	B2	Argentina	Bovine	✔	✔
*Tritrichomonas augusta*	30082	Czec Republic^1^	Frog	✔	✔
*Tetratrichomonas gallinarum*	MR5	Czec Republic^1^	Chicken	✔	✔
*Trichomonas gallinae*	TG09	Porto Alegre/Brazil	Pigeon	✔	
*Trichomitus bathracorum*	G43	New York City/USA	Snake	✔	
*Monocercomonas sp*	-	Cuba	Snake	✔	

✔ Samples used in each hybridization experiments;

Isolated by: ^1 ^J. Kulda (Charles University in Prague); ^2 ^H. Guida (Embrapa).

**Table 5:** List of oligonucleotide primers used in this study

**Primer**	**Sequence**	**Positions (bp)**	**Expected length** **(bp)**
TvMULE1_F	5'-AAGCGAGCATGAACTGCATCA	229–249	696
TvMULE1_R	5'-TTCCGATCAAGGTCCGGCAATTA	902–924	
TvMULE2_F	5'-GCTGACTGTGCGCTAAACATTGCT	1055–1078	544
TvMULE2_R	5'-GCTCAACAATCTGATTACCTGCCC	1575–1598	
TvMULE3_F	5'-GGGTATCAAAGAACAAGAGTCACC	1,286–1,309	630
TvMULE3_R	5'-TCTCTTTCAGCGGCTGTCCATCTT	1,892–1,915	
TvMULE4_F	5'-GGACAAACTCGCCCATTGTCACTT	8–31	584
TvMULE4_R	5'-TCTTGACAGGTGGATGCTTCGCTA	568–591	
TvMULE4_2F	5'-TTCGCCTTTCTGGGAAGTACTGGT	485–508	520
TvMULE4_2R	3'-GTCACTGGCAAATTCGCGGAATCA	981–1,004	
β tubulin_F	5'-ACACTCCTTCTCAACAAGCTCCGT	692–715	673
β tubulin_R	5'-AGGCTGTTGTGTTGCCGATGAATG	1341–1364	

To verify if the *TvMULEs *are transcriptionally active, polyA^+ ^RNA was extracted and cDNAs synthesized from one strain from *T. vaginalis *(JT) and six non-*T. vaginalis *species and isolates (Table 4). Again, RT-PCR products were obtained for each sample using the primer pairs of each element and their homology to *TvMULEs *validated by hybridization using the sequence from the JT strain of *T. vaginalis *as probe. The presence of abundant mRNA for the four *TvMULEs *was observed in the JT strain (Figure 5B), confirming that the four *Mutator *elements are active transcriptionally in *T. vaginalis*. In contrast, the other species show no evidence of transcripts of the expected size (Figure 5B).

## Discussion

Transposable elements are major players in the evolution of eukaryote genomes. *T. vaginalis*, whose two-thirds of the genome consists of repetitive sequences, is a fascinating species to study in this context, since several topics can be explored: the discovery of new TEs, their structure and origin, the dynamic of TEs among related species and geographical populations, and their comparison to those characterized in other fully sequenced genomes. *Mutator *elements are one of the most thoroughly studied plant TEs [21,37,38,40-42,44,46,56-62]. For nearly three decades after their initial discovery by Robertson [35] they were thought to be present exclusively in plants. The first homologous representatives were completely characterized in the early 2000's in fungi [22,23] and in the amoebozoa [24]. We have conducted a comprehensive study of four new members of the *Mutator *superfamily in a new taxonomic group, the Parabasalids, and in particular the class Trichomonada, and conclude that three of the elements found are representatives of a new branch in the evolutionary history of the *Mutator *superfamily.

This study shows that only *TvMULE1 *is a typical member of the *Mutator *superfamily, since it shows significant similarity to *Mutator *proteins with known transposase motifs and harbors some of the hallmarks of *MULEs*. Interestingly, *TvMULE2*, *TvMULE3 *and *TvMULE4*, in addition to the presence of a conserved *Mutator*-like transposase domain and a motif identical to the 25-aa signature sequence of the *MULE *transposase core, also display new and distinct conserved motifs. The presence of *Mutator*-like elements in Trichomonads is not unrealistic, as the evolutionary relatedness between the maize *Mutator *autonomous elements and the bacterial *IS256 *[21] shows this superfamily's ability to invade hosts across large evolutionary distances or to survive, by vertical transmission, across the spectrum of life. New *MULE *families have already been characterized in other early divergent eukaryotes, such as in the first genomes analyzed from the genus *Entamoeba *[24]. What is perhaps surprising is that it took over two decades for elements of the *Mutator *superfamily to be identified in eukaryotic taxa other than plants. Our Southern blot experiments using *TvMULE *probes strongly suggest their presence in other trichomonad species and our *in silico *analyses allowed their identification in the *C. albicans *genome.

Elements similar to our repeats *TvMULE2*, *TvMULE3 *and *TvMULE4 *have been submitted to Repbase Reports, namely *MuDR-4_TV *[63], *MuDR-3_TV *[64], *MuDR-5_TV *[65], respectively. These repeats and their structures differ somewhat from those found here described in one or more of the following characteristics: (1) length of the elements and the peptides they encode; (2) length of TSDs; and (3) copy number estimates. The differences could be due to the methods employed to determine the canonical consensus sequences.

The four *TvMULEs *each carry a putative transposase ORF, which are smaller than those of known *MULE *Tpases but seem, nevertheless, to be functional since independent lines of evidence support their transpositional activity. The level of sequence divergence between copies and their respective consensus sequences (identity >99%) and the presence of complete copies inserted in different scaffold locations suggest that these families have undergone a recent process of activation and amplification. In addition, the set of expressed mRNAs includes transcripts with high sequence similarity to these repeats. Interestingly, typical *MULE *TIRs, characteristically over 100 bp long and the perfect inverted complement of each other, and which are supposedly necessary for mobilization, were not identified in *TvMULES*. We hypothesize that these repeats represent of a novel type of non-TIR-*MULE*s, similar to those identified in *A. thaliana*, which are able to transpose in the absence of long TIRs [40].

The large number and mobility of *TvMULEs*, much like those observed for other TEs already characterized in *T. vaginalis *[9,50-52], raise puzzling questions. What are the biological and epidemiological features that explain such high level of recent transposon activity in *T. vaginalis*, while these elements present a heterogeneous distribution among other Trichomonads examined? Could these elements have been recently introduced into *T. vaginalis *and, if so, where from? How do these TEs contribute to the architecture and dynamics of this highly repetitive genome? What in the *T. vaginalis *genetic background makes this genome permissive to the high activity of these DNA transposons, to the extent that they have accumulated to hundreds and even thousands of copies per family [9]?

A fascinating hypothesis to explain the extraordinary expansion of TEs in the genome of *T. vaginalis *was proposed by Carlton and collaborators [9]. *T. vaginalis*, unlike most other Trichomonads which are enteric, is a parasite of the human urogenital tract. A large cell size is likely advantageous in this species, since it increases its phagocytosis ability, decreases the probability of it being ingested by other organisms and host macrophages, and facilitates adhesion to vaginal epithelial cells. There is a strong, and possibly causal, correlation between genome size and cell size [66-68]. Therefore, an initial stochastic expansion of TE families could have given rise to the variation upon which natural selection could act, favoring the largest cells and, concomitantly, those with the largest TE complement [9]. It is interesting to note that *Tritrichomonas foetus*, the only other vaginal trichomonad surveyed, was the only other species in which all four *TvMULEs *were detected.

The large copy number and extremely low polymorphism of *TvMULE*s and other *T. vaginalis *repeats, as well as their absence in *T. tenax*, a parasite of the bucal cavity and the sister taxon to *T. vaginalis*, suggest a fast repeat expansion that has taken place in a recent evolutionary past [9]. The lack of homologs of the *T. vaginalis *repeats in *T. tenax *[9] also raises the possibility that these elements have been recently acquired through horizontal transfer, a phenomenon that is relatively more common than was once believed, and which is possibly an essential step in the life-cycle of successful class II transposable elements [69,70]. Here we found evidence for the presence of some *TvMULE *homologs in some of the species surveyed. In particular, only *TvMULE4 *shows a strong hybridization signal in *T. gallinae*, the closest species to *T. vaginalis *examined in this study, while homologs to the other three *TvMULE *families are present in more distantly related species. The possibility remains that these repeats could have been lost from some species, or that the PCR primers used did not amplify existing divergent homologous repeats, an issue that can only be solved with an extensive genomic survey of the family Trichomonadidae.

Transposable elements have undeniably played a major role in the expansion of eukaryotic genomes, a phenomenon well documented in plants [71], arthropods [72] and vertebrates [73-76]. Rapid genome expansions due to bursts of TE amplification, similar to what is observed in *T. vaginalis*, have also been postulated for a variety of organisms [77-81]. What sets *T. vaginalis *apart is the fact that it is an asexual species, which, like all other trichomonads, reproduces by longitudinal binary fission. It has been argued that transposons are unable to persist in the long term in clonal lineages because the mechanisms that keep TE copy number in check in sexual species, and that thereby prevent excessive mutational loads, are absent in asexual lineages [82]. In addition, once lost, they cannot be reintroduced by sexually-mediated genetic transfer [83]. Given the recency of the TE expansion in *T. vaginalis*, their long-term effect on the survival of the species is as yet unclear. It is possible that, with each TE family expansion, this species is steadily proceeding to extinction.

## Conclusion

The remarkably recent common ancestry of each TE family in the *T. vaginalis *genome is attested to by the high copy number and nearly complete within-family sequence similarity of these *TvMULEs*, features that are shared with the other ~55 repeat families identified in the *T. vaginalis *genome. The structure of each repeat, inferred from the consensus of all copies within a family, is therefore likely to reflect with high accuracy the ancestral sequence of each original active element. This makes the genome sequence of *T. vaginalis *is an ideal mining ground for new transposable elements, which sequence and structure have not yet been adulterated by the accumulation of inactivating mutations.

## Methods

The consensus sequences of the newly characterized *Mutator*-like elements from *Trichomonas vaginalis *described here have been submitted to Repbase Reports .

### *In silico *analyses

The draft genome sequence of the G3 strain of *T. vaginalis *was obtained from the website of The Institute for Genomic Research (TIGR) . This draft, based on ~7.2-fold coverage of the genome, consists of 17,290 scaffolds, representing ~160 Mbp [9]. Sequence similarity searches using the four consensus sequences of *TvMULEs *as query against the *T. vaginalis *genome were performed using BLASTN [84], with parameters E = e-20, V = 10,000 and B = 10,000. Significant matches were required to be >200 bp long and display ≥ 80% identity. We will refer to the repeat copies found in the genomes according to the contig scaffold name and the start and end position of the copy. The coordinates of each BLASTN match were extracted using our customized Perl scripts, which utilized some modules of the BioPerl toolkit [85], and aligned with ClustalW [86] with default parameters. When available, the regions flanking each insertion were extracted for additional analyses: i) logo sequences were built from the first 25 nt upstream and downstream of each insertion using WebLogo [87], ii) the extent of the similarity between insertions, in regions upstream of the 5' end and downstream of the 3' end, was evaluated by BLASTN, and iii) the "guanine and cytosine" content (percent GC) was calculated from the first 100, 2,000 and 5,000 flanking nucleotides using the program "geecee" of the EMBOSS package .

As *T. vaginalis *genes are mostly intronless all open reading frames (ORFs) corresponding to protein coding genes start with a methionine (Met) residue. The location of all ORFs starting with a Met residue that were at least 100 amino acids in length was determined for all contigs that contained the four *TvMULEs*, using the program "getorf" of the EMBOSS package. Homologs to the most frequent ORFs associated with each TE were detected by BLASTP against the non-redundant protein database in GenBank. Conserved domains were predicted with the « Conserved domain search » toolbox from NCBI [88] or the MEME package [89]. The putative occurrence of conserved terminal inverted repeats (TIRs) was analyzed by BLAST 2 sequences [90] and manual inspection.

### Phylogenetic Analyses

Additional sequences of *Mutator *elements and related TE families from a variety of taxa, including plants, fungi, protists and bacteria, were obtained from GenBank, Repbase Report, TIGR  and the BLAST Server of the Sanger Institute . Highly conserved regions in 56 protein sequences of *Mutator *and *IS256 *were detected using MEME, with the following parameters: number of different motifs = 15; minimum and maximum motif width = 5 and 300 amino acids, respectively. Twelve motifs were identified, of which *motif 1 *is conserved in all sequences, *motif 8 *occurs with the second highest frequency followed by *motif 4 *[see Additional file 2]. These three motifs are contiguous in the following orientation: motif 4 → motif 8 → motif 1. The sequences with motifs 4 and 8 were used as reference for discovering homologous regions by manual inspection in proteins where they were not identified by MEME due to their higher sequence divergence. The three motifs were found in 48 of the initial 56 sequences. This region containing motifs 4, 8 and 1 was extracted and aligned by CLUSTALW [86] with default parameters; the alignment was refined manually [see Additional file 3]. Two methods were used to reconstruct the evolutionary relationships among the sequences: i) *neighbor-joining *(NJ) with the JTT substitution model, pairwise deletion condition and the bootstrap analysis consisted of 1,000 replicates as implemented in MEGA4 [91], and (ii) a bayesian analysis, implemented in MrBayes v3.1.2 [92]. Model settings for MrBayes were as follows: amino acid transition matrix was set to a mixture of models with fixed rate matrices (Poisson, Jones, Dayhoff, Mtrev, Mtmam, Wag, Rtrev, Cprev, Vt, and Blosum) of equal prior probabilities, site rate variation described by a gamma distribution (*α *uniformly distributed between 0–200, with 4 rate categories), and a proportion of invariant sites uniformly distributed between 0.0–1.0. Branch lengths were unconstrained and described by an exponential distribution (10.0). Two simultaneous runs of MrBayes, with 4 chains each, ran for 1,500,000 generations. Results were evaluated after a burn-in period of 10% (150,000 generations) and convergence was achieved (PSRF= 1.00) for all model parameters estimated, including tree length (mean = 18.8), *α *= 2.28 and the proportion of invariant sites (4%), the amino acid model (Blosum), and the tree topology (see results).

### Trichomonad species and Culture medium

The trichomonad species used in this study are listed in Table 4. Cultures were maintained in TYM Diamond's medium [93] as suggested by the American Type Culture Collection (ATCC), and grown at 36.5°C until reaching 5 × 10^6^cells. The samples were collected by low speed centrifugation and washed two times in phosphate-buffered saline (PBS, pH 7.2).

### DNA amplification and sequencing

Amplification of each of the four *TvMULEs *was performed with primer sets designed to amplify an internal region of the transposase domain (Table 5). PCR was done in a volume of 25 μl with 0.5U of Taq DNA polymerase in 1× polymerase buffer, 10 μM of each primer, a 200 μM concentration of each dNTP and 1.5 mM MgCl_2. _The solutions were heated to 94°C for 2 min, and followed by 35 cycles of denaturation (94°C for 1 min), annealing (60°C for 2 min), and extension (72°C for 1 min), followed by a final extension at 72°C for 10 min. PCR products with the expected size were excised from 1% agarose gels, purified using GFX™ PCR DNA and Gel Band Purification Kit (GE Healthcare, Little Chalfont, UK), and cloned using TOPO TA Cloning Kit (Invitrogen, Carlsbad, CA). To confirm the identity of the PCR products from the *T. vaginalis *JT isolate, both strands of two clones for each transposon, chosen at random, were sequenced using the BigDye Terminator mix (Applied Biosystems, Foster City, CA) and run on an ABI 377 sequencer (Applied Biosystems, Foster City, CA). The clones were used as probes to confirm DNA and cDNA PCR amplification of each *TvMULE*.

### DNA and cDNA hybridization analyses

Genomic DNA was extracted from the eight trichomonad species listed in Table 4 using DNAzol^® ^reagent (Invitrogen, Carlsbad, CA), and PCRs run on each sample with *TvMULE*-specific primers. The occurrence of *TvMULEs *in different species was confirmed by Southern blot of PCR products using the detection system *Gene Images *CDP-Star detection module (Amersham Biosciences, Little Chalfont, UK), due to non-availability of total DNA content sufficient for direct DNA gel blot. Cloned *TvMULE *transposase fragments were labeled with the chemioluminescent hybridization system *Gene Images *random-prime labeling module (Amersham Biosciences, Little Chalfont, UK). PCR products were separated in 1% agarose gels and transferred to Hybond N+ membranes (Amersham Biosciences, Little Chalfont, UK). Blots were prehybridized 1 h at 60°C in 5× SSC, 5% dextran sulfate and 20-fold dilution of liquid block and hybridized overnight with the probes of each *TvMULEs*. Blots were washed twice with 0.2× SSC, 0.5% SDS and exposed to autoradiographic film for 20 minutes at room temperature.

In order to identify transcriptional activity, PolyA+ RNA was isolated from total RNA of each species listed in Table 4 using TRIzol reagent (Invitrogen, Carlsbad, CA). 5 μg polyA+ RNA was used for cDNA synthesis using High Capacity cDNA Reverse Transcription kit (Applied Biosystems, Foster City, CA) with random primers and Oligo d(T)12 (Gene Link™, Hawthorne, NY) at low stringency (37°C). RT-PCR products of each cDNA sample were electrophoresed on 1% agarose gels, and the fragments were transferred onto Hybond N+ membranes. Prehybridization, hybridization, washing and detection were performed as for DNA hybridization.

## Abbreviations

TEs: Transposable elements; TIRs: Terminal inverted repeats; MULEs: *Mutator*-like elements; TSDs: Terminal site duplications; *TvMULEs*: *Trichomonas vaginalis Mutator*-like elements; Tpase: Transposase; NJ: neighbor-joining; ORFs: open reading frames.

## Authors' contributions

FRL conceived the project and wrote the manuscript and was responsible for data collection, analyses and interpretation. JCS performed the bayesian analysis, participated in data interpretation and writing. MB provided DNA samples of trichomonad species and strains as well as laboratory facilities for the organism culture and mRNA extraction. GGLC and GAGP provided the PERL scripts and final style of the figures 1, 2, 3, and performed BLAST runs. CMAC coordinated the project, participated in data interpretation and the manuscript elaboration. All authors read and approved the manuscript.

## Supplementary Material

Additional file 1 **Conserved motif in the transposases of elements from the *Mutator*-*IS256 *superfamily**. A – Multiple alignment of *motif 1 *with 36-aa. B – Sequence logo. The vertical axis has a maximum value of 4 and is proportional to the level of sequence conservation at each position. Identical residues or those sharing similar physical or chemical properties are shown in black if present in all sequences, and in gray if present in the majority of the sequences. Each sequence name contains the species or TE (if previously assigned) name, the gi accession number and the coordinates of residues included in the alignment. Click here for file

Additional file 2 **Summary of 12 motifs identified by MEME in 56 proteins of Transposase from *Mutator *and *IS256 *superfamily**. The protein length is shown in the bar scale, except those for which the length is annotated on the right. Click here for file

Additional file 3 **Clustal alignment of the domain found in the transposases from the *Mutator *– *IS256 *superfamily**. Five main clades and the region of the three conserved motifs are shown. Click here for file
