# Supplementary material for: The protist Trichomonas vaginalis harbors multiple lineages of transcriptionally active Mutator-like elements
Source: BMC Genomics. 2009 Jul 21;10:330. doi: 10.1186/1471-2164-10-330 (PMC2725143; doi:10.1186/1471-2164-10-330)

A

Facid_48851616:217-252	DGIKNLDEEVMEIYPRSEFQLCTIHYARGLKSNVRE
Ccell_27881402:228-263	DKCLGMLESVNEVFPEAKYQRCTVHFYRNIFSVTPR
Efaec_29377857:233-268	DAHKGLVSAIRKSFTNVSWQRCQVHFLRNIFTTIPK
Sepid_13383310:233-268	DAHKGLVSAIRKSFTNVSWQRCQVHFLRNIFTTIPK
Mmaze_21228632:242-277	DGHKGIQKAVRESFIGSSWQMCHVHLIRQALKKVQK
Bline_6456721:243-278	DAHSGIKAAVKAILPGAGWQRCRVHFARNVTQRLGS
Mtube_15610776_233-268	DQHAGLVKALKRCFQGAGHQRCRVHFARNLLAHVPK
Maviu_4210728:232-267	DAHTGLRSAIEAILIGASWQRCRVHFLRNVLAQVPK
Ehist_284.m00070:394-429	DRCIAQYNALKLLFPYSKLFFCRIHIERSLKKYFKN
Ehist_c000407510:200-235	DRCIAQYNALKLLFPHSKLFFCRIHIERSLKKYFKN
Ehist_c000407727:200-235	DRCIAQYNALKLLFPYSKLFFCRIHIERSLKKYFKN
Edisp_167379831:282-317	DRSIAQYNALKHVFPHSKLFFCRIHIERSLIKYFKS
Emosh_004g04.qlk:61-96	DRSNAQFRAIQTVFPNSEIIFCKVHIQRSIEKYFHN
Emosh_010h03.plk:98-133	DRSNAQFRAIQTVFPNSEIIFCKVHIQRSIEKYFHN
Emosh_131h06.qlk:214-249	DRSNAQFRAIQTVFPNSEIIFCKVHIQRSIEKYFHN
Emosh_117b06.plk:146-181	DRSNAQFRAIQTVFPNSEIIFCKVHIQRSIEKYFHN
EMULE_Eil:279-314	DRCLSQTNALLQVFKDVNLVFCTRHIRRNLITEFGK
Einva_035b12.qlk:154-189	DRCFSQTKALETSFNGISIFHCVRHIRRDLQVAFGK
Hop_30421204:298-333	DRCLACMNAVSSCFPGSALFLCLWHINKAVQSYCRP
Cglob_11619630:301-336	DRCIACMNAVSTCFPSAASLLCLWHANKAILRHCQP
Mtrun_124361025:272-307	DRDMSLMKAVAHVFPESYALNCFFHVQANVKQRCVL
Cneof_58264436:296-331	DRDPALINALMSVLPKAYRFSCFWHLQENVKSNIRP
Ptrit_189208826:332-367	DHDRSLKAGLSVIFPGIPQRRCIWHIYQNVQTEAVK
Zmays_19908843:325-360	DQDPAMPIALRTVFPKTVHRLCLWHVQNRFMPFLNE
Osati_50904427:646-681	DQDLAMRAAIRQVFPNSKHRNCLFHILKKCRERSGN
Athal_5764395:341-376	DQDKFLMSAVSELLPNTRHCFALWHVLEKIPEYFSH
Vvini_157338603:444-479	DQDKALKEAIAEVFPESRHCFCLWHILSKIPEKLSC
Jittery_7673677:310-345	DEDASIKSAIRTTLPDTIHRLCMWHIMEKVSEKVGH
Osati_50931191:1,002-1,037	DEDASMKAAIAQILPNTVHRLCMWHIMEKVPEKVGP
MURAZC_23928448:166-201	DACKGLKNAMNNVFPHAEKRECFRHLIQNYIKLFGG
MudrA_540581:393-428	DAQKGLMHAVNEVFPYAERRECFRHLMGNYVKHHAG
Osati_37700327:544-579	DKQKGLIPAVQQLFPDSEHRFCVRHLYQNFQQSFKG
Osati_34914922:481-516	NKQKGLIPAVKKVFPDTEHRFCVRHLYSNFQEKFKG
**TvMULE1: 340-375**	DQGPAIIAAVRESGFSCDHKFCLRHFATKREYINVY
Athal_11994228:486-521	DRNSSLIKAVAEVYPSSHHGNCVYHLSQNVRTKVAY
Athal_8777291:593-628	DRHESIKVGVKKVFPQAHHGACIIHLCRNIQARFKN
Athal_15225608:540-575	DRHNSVYASIRKVYPMSSHAACVVHLKRNIEASFKY
Athal_22331509:653-688	DRNQSIFKVVSLVFNQAHHGACLVHIRRNVKGRYVI
Athal_15224361:640-675	DRHSSIYKGVSVVYPKANHGACIVHLERNISVSYAR
Athal_4220448:592-627	DRHSSIYKGLSVVYPRAHHGACAVHLERNLSTYYGK
**Calbi_68474652:182-217**	DADPAEISGIQSIFKDTKIVLCYFHVLRAVTIKLKE
**Calbi_68466277:370-405**	DCSMPELSAIKTVFPESSVSICKWYILRNVRTEARS
**Calbi_68466572:112-147**	DCSMSELSAIKTVFPESSVSICKWHILRNVRTEARS
**TvMULE2: 262-297**	DCALNIARAIEDGFPLAQLSWCAVHVLRACAKVGSY
**TvMULE3: 52-87**	DCAQEIENAIINSFPEVILHWCAVHVMRAFRKNLKD
**TvMULE4: 255-290**	DCAPQITNAVETAIPLCQIIWCGVHVLRAVMRKAEK

B

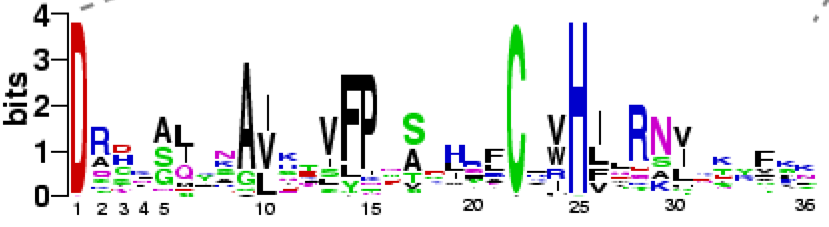

Supplement: Additional file 1 — Conserved motif in the transposases of elements from the Mutator-IS256 superfamily. A – Multiple alignment of motif 1 with 36-aa. B – Sequence logo. The vertical axis has a maximum value of 4 and is proportional to the level of sequence conservation at each position. Identical residues or those sharing similar physical or chemical properties are shown in black if present in all sequences, and in gray if present in the majority of the sequences. Each sequence name contains the species or TE (if previously assigned) name, the gi accession number and the coordinates of residues included in the alignment. [file 1471-2164-10-330-S1.pdf]
