# Supplementary figures and images for: The protist Trichomonas vaginalis harbors multiple lineages of transcriptionally active Mutator-like elements
Source: BMC Genomics. 2009 Jul 21;10:330. doi: 10.1186/1471-2164-10-330 (PMC2725143; doi:10.1186/1471-2164-10-330)

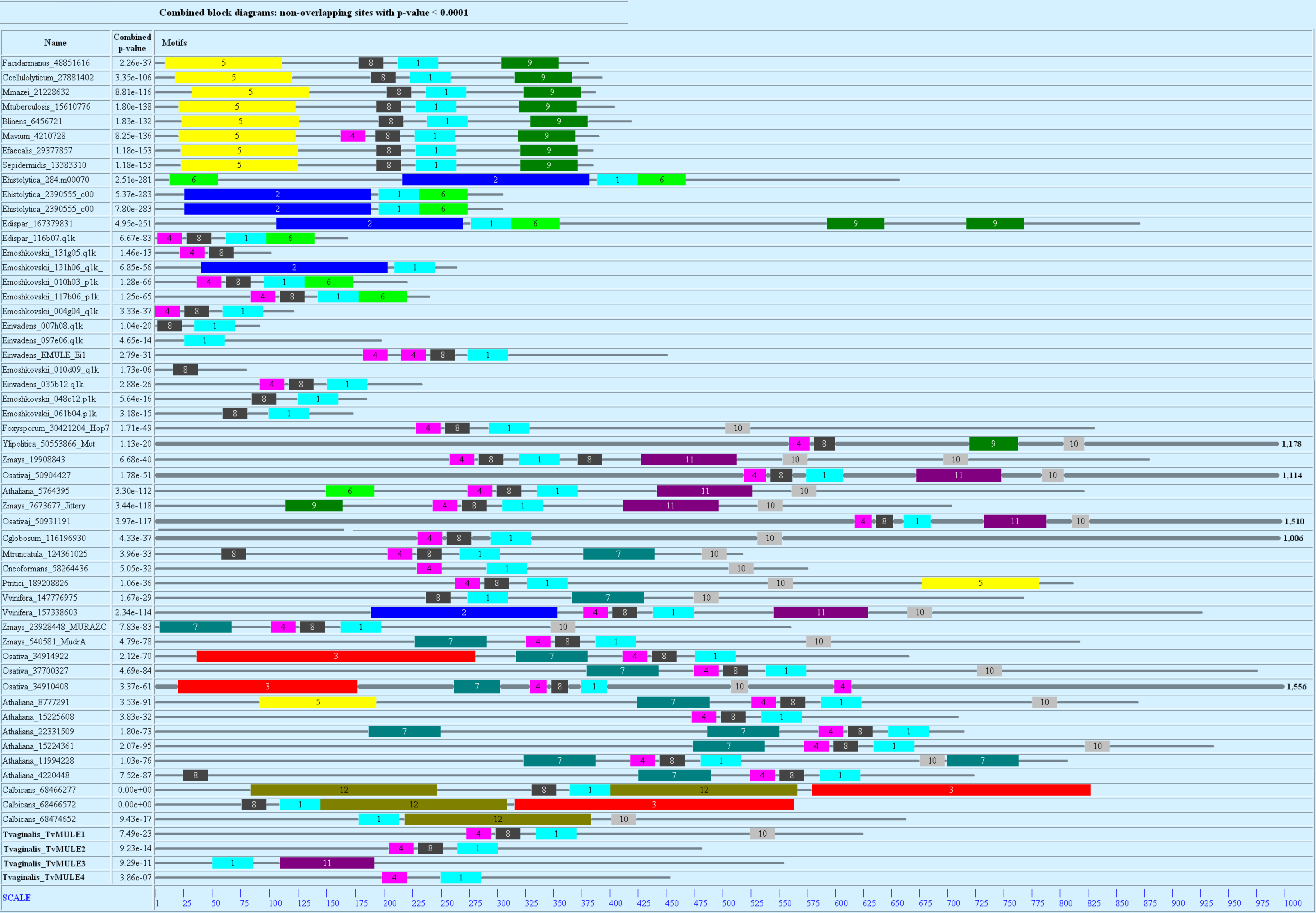

Supplement: Additional file 2 — Summary of 12 motifs identified by MEME in 56 proteins of Transposase from Mutator and IS256 superfamily. The protein length is shown in the bar scale, except those for which the length is annotated on the right. [file 1471-2164-10-330-S2.pdf]
