# Supplementary material for: The protist Trichomonas vaginalis harbors multiple lineages of transcriptionally active Mutator-like elements
Source: BMC Genomics. 2009 Jul 21;10:330. doi: 10.1186/1471-2164-10-330 (PMC2725143; doi:10.1186/1471-2164-10-330)
Supplement: Additional file 3 — Clustal alignment of the domain found in the transposases from the Mutator – IS256 superfamily. Five main clades and the region of the three conserved motifs are shown. [file 1471-2164-10-330-S3.pdf]

| Family | Sequence | Alignment |
|---|---|---|
| | | `         10        20        30        40        50        60` |
| | | `....\|....\|....\|....\|....\|....\|....\|....\|....\|....\|....\|....\|...` |
| *IS256* | Facidarmanus_48851616 | `DGLFFYLR----RGNVDKEPVIFALGIKET--GEYEVLGFYLT-VKESHNAYKEVLEDLYN--` |
| | Ccellulolyticum_27881402 | `DGIYLKRN---WGGEYENVSILVAIAVNQE--GYREVIGAREG-MKEDKASWKEFLQWLKG--` |
| | Mmazei_Go1_21228632 | `DATYLKVR---DGLHYENKALFIVSGVRDD--GFREILGARLA-DSEDSLFWQDLFEDLKE--` |
| | Mtuberculosis_H37Rv_15610776 | `DATYLNVRN--GTGQVVSMAVIVASGIAAD--GSREILGLDVG-DSEDETFWRGFLTSLRE--` |
| | Blinens_6456721 | `DATYVDVR---HRGRVVSQAVAVVTGVSSQ--GRREILTMSVG-DAESTDFWTQVLRGLKT--` |
| | Mavium_4210728 | `DATYCKAR---VNHRVVSQAVVIATGVAAD--GRREVLGFDVG-DSEDGAFWTAFLRSLKE--` |
| | Efaecalis_V583_29377857 | `DVLYIKVR---EENRVLSKSCHIAIGITKD--GDREIIGFMIQ-SGESEETWTTFFEYLKE--` |
| | Sepidermidis_13383310 | `DVLYIKVR---EENRVLSKSCHIAIGITKD--GDREIIGFMIQ-SGESEETWTTFFEYLY---` |
| *EMULEs* | Ehistolytica_284.m00070 | `DDTAKTNI--------YNKNLYVIIVKDDN--SFNQLLSFGYL-FDQSECSYKFFLHQLY---` |
| | Ehistolytica_2390555_c407510 | `DDTAKTNI--------YNKNLYVIIVKDDN--SFNQLLSFGYL-FDQSECSYKLFLHQLY---` |
| | Ehistolytica_2390555_c407727 | `DDTAKTNI--------YNKNLYVIIVKDDN--SFNQLLSFGYL-FDQSECSYKFFLHQLH---` |
| | Edispar_167379831 | `DDTAKTNI--------YNKNLYAVIVKDDN--SFNQFLSFGYL-FDQSENSYKLFLHQLN---` |
| | Emoshkovskii_mosh131h06.q1k | `DDTVGLTK--------YHKPVQIISTIDEC--HRTQILSYGIT-NNQTTDSYFSYFKQIN---` |
| | Emoshkovskii_mosh010h03.p1k | `DDTVGLTK--------YHKPVQIISTIDEC--HRTQILSYGIT-NNQTTDSYFSYFKQIN---` |
| | Emoshkovskii_mosh117b06.p1k | `DDTVGLTK--------YHKPVQIISTIDEC--RRTQILSYGIT-NNQTTDSYFSYFKQIN---` |
| | Emoshkovskii_mosh004g04.q1k | `MTLVGLTK--------YHKPVQIISTIDEC--HRTQILSYGIT-NNQTTDSYFSYFKQIK---` |
| | Einvadens_EMULE_Ei1 | `DDTCGTNE--------FGLPLVVAVNIDKE--KRSHCVFFALM-TNRTTQSFVLVFSYVKS--` |
| | Einvadens_inv035b12.q1k | `DDTAATNT--------FNFPLVIGVFSDVH--NKTKVLFFSIL-RNRTTESFIDLFEFLR---` |
| *Hop/Jittery* | Foxysporum_30421204_Hop78 | `DSTYKTNR--------FKMPLLDIVGVDAC--QRTFCIAFAFL-SGEEEGDFTWALQALK---` |
| | Ylipolitica_50553866_Mutyl | `DVTHNTNK--------FNYKLFNVIGIASC--GRSFNIAHALI-SREDADTFKWCLDRLK---` |
| | Zmays_19908843 | `DTTHKTNI--------YDKPLGMFVGANSH--LQCTVFGFVLL-GDETVQTFEWAFNSFL---` |
| | Osativa_50904427 | `DTTYKTNR--------YNMPFAPIVGVTGH--GNICIFACAFL-GDETTETFKWVFETFL---` |
| | Athaliana_5764395 | `DTTYVKFN--------DKLPLALFIGVNHH--SQPMLLGCALV-ADESMETFVWLIKTWL---` |
| | Zmays_7673677_Jittery | `DATYSTNQ--------YNMRFAPFTGVNHH--MQRVFFGAAFL-ANEKIESYEWLFRTFL---` |
| | Osativa_50931191 | `DSTYSTNQ--------YNMKFVPFTGVNHH--LQSVFLGASFL-ADEKIESFVWLFQTFR---` |
| | Cglobosum_116196930 | `DCTYKTNN--------YGMPLLDMIGVDAC--QRSFCIAFAFL-HGETEEDYCWALDQLR---` |
| | Mtruncatula_124361025 | `DSTYKTNM--------YRMPMFEVVGVTST--DLTYSVGFGFM-THEKEENFVWVLTMLF---` |
| | Cneoformans_58264436 | `DCTYKTNL--------YRMPMLHIVGSTST--GMTYTAGVILM-LRETTNWYTQALNSFK---` |
| | Ptritici_189208826 | `DATYKTNA--------FNMPLVHIVGITCR--ATTYDIAYAFI-PNEAAETYLEVVQYLK---` |
| | Vvinifera_157338603 | `DTTYIKNE--------YKLPFAPFIGVNHH--FQFVLLGCALI-ADETKSTLVWLMRSWL---` |
| *MuDR* | Zmays_23928448_MURAZC | `DSTALNGR--------WNGHLCCAIGVDGH--NWMYPVAYGFF-EAENTENWTWFFHQLHK--` |
| | Zmays_540581_MudrA | `DSTALNGR--------WNGHLASATGVDGH--NWMYPVCFGFF-QAETVDNWIWFMKQLKK--` |
| | Osativa_34914922 | `DGCHIKTK--------FGGKLLTAVGMDPN--DCIFPIAMAVV-EVESFVSWEWLLETLKSEL` |
| | Osativa_37700327 | `DGCHIKTK--------FGGQLLTAVGIDPN--DCIFPIAMAVV-EVESFSTWSWFLQTLKDDV` |
| | Athaliana_8777291 | `DGTHLKGK--------YKGVLLTASGQDAN--FQVYPLAFAVV-DSENDDAWTWFFTKLER--` |
| | Athaliana_15225608 | `DGTHLRGR--------YGGYLVAASAQDAN--FQVFPIAFRIV-NSENDEAWTWFMTKLTE--` |
| | Athaliana_22331509 | `DGTHLFGK--------FLGCLLTASCQDAN--FQILPIAFAVV-DSETNESWSWFMNKLSE--` |
| | Athaliana_15224361 | `DGTQLVGR--------YKGCLLIACAQDGN--FQIFPLAFGVV-DGETDASWIWFFEKLSE--` |
| | Athaliana_11994228 | `DATFLKTI--------YKGVLIFATAQDPN--HHHYPLAFAVA-DGEKDVTWKWFFETLKT--` |
| | Athaliana_4220448 | `DGTQLVGP--------YKGCLLIVCAQDGN--FQIFPIAFGVV-DGETDASWAWFFEKLAE--` |
| | Osativa_34910408 | `DGTFLTGK--------YQGTLLTAIGVDAG--LHLVPLAFALV-EKENTSNWEWFINMLRNK-` |
| | **Tvaginalis_TvMULE1** | `DGTFLFCI--------KKGNLL-IIGTPAPN-NRLIPIAFAWS-VSENTITIKDMLTKLKS--` |
| *TvCaMULEs* | **Calbicans_68466277** | `DFGKRMKN--------SSVYLYTVVARDKVTG-MGVPCAFFIT--NTLKEDPIISFLGFLKS-` |
| | **Calbicans_68466572** | `DFGKRMKN--------SSVYLYTVVARDKVTG-MGVPCAFFIT--NTLKEDPIISFLEFLKS-` |
| | **Calbicans_68474652** | `DATHGLVKSLN---GQSNAYLFVLTGIIPSS-RNTFPLSFMLT-NYTGKITIQHWLNNLK---` |
| | **Tvaginalis_TvMULE2** | `DSTHSLIK--------GKIQLFAVTMKTSQN--TIFPFCYFL-VNPQTSEKIQECLVKFCEF-` |
| | **Tvaginalis_TvMULE3** | `------------------MLFYMISAKFPNT--HAFPIFQFV-VYPNTSENIAFCLKAFFNW-` |
| | **Tvaginalis_TvMULE4** | `DSTYKLLR--------SRIPFYAVTGKFAES------------IGPDTSENIQVCLTAYFGS-` |

Motif 4 (columns 1–30) · Motif 8 (columns 37–61)

*Continued*

| Group | Sequence | 70 80 90 100 110 120 |
|---|---|---|
| ***IS256*** | Facidarmanus_48851616 | -RGLK--------EPLLIVADGIKNLDE-EVM---EIYPRSEFQLCTIHYARGLKSNVRE |
| | Ccellulolyticum_27881402 | -RGLD--------GVKLIVGDKCLGMLE-SVN---EVFPEAKYQRCTVHFYRNIFSVTPR |
| | Mmazei_Go1_21228632 | -RGLR--------GVKLIVSDGHKGIQK-AVR---ESFIGSSWQMCHVHLIRQALKKVQK |
| | Mtuberculosis_H37Rv_15610776 | -RGLG--------GVRLVISDQHAGLVK-ALK---RCFQGAGHQRCRVHFARNLLAHVPK |
| | Blinens_6456721 | -RGLKVSTETDPEGVALVISDAHSGIKA-AVK---AILPGAGWQRCRVHFARNVTQRLGS |
| | Mavium_4210728 | -RGLS--------GVQLVISDAHTGLRS-AIE---AILIGASWQRCRVHFLRNVLAQVPK |
| | Efaecalis_V583_29377857 | -RGLQ--------GTELVISDAHKGLVS-AIR---KSFTNVSWQRCQVHFLRNIFTTIPK |
| | Sepidermidis_13383310 | -RGLQ--------GTELVISDAHKGLVS-AIR---KSFTNVSWQRCQVHFLRNIFTTIPK |
| ***EMULEs*** | Ehistolytica_284.m00070 | -NILNY-------TPQIIVCDRCIAQYN-ALK---LLFPYSKLFFCRIHIERSLKKYFKN |
| | Ehistolytica_2390555_c407510 | -NILNY-------TPQIIVCDRCIAQYN-ALK---LLFPHSKLFFCRIHIERSLKKYFKN |
| | Ehistolytica_2390555_c407727 | -NILNY-------TPQIIVCDRCIAQYN-ALK---LLFPYSKLFFCRIHIERSLKKYFKN |
| | Edispar_167379831 | -NILNY-------GPEIIVCDRSIAQYN-ALK---HVFPHSKLFFCRIHIERSLIKYFKS |
| | Emoshkovskii_mosh131h06.q1k | -EYLN--------SPDIIICDRSNAQFR-AIQ---TVFPNSEIIFCKVHIQRSIEKYFHN |
| | Emoshkovskii_mosh010h03.p1k | -EYLN--------SPDIIICDRSNAQFR-AIQ---TVFPNSEIIFCKVHIQRSIEKYFHN |
| | Emoshkovskii_mosh117b06.p1k | -EYLN--------SPDIIICDRSNAQFR-AIQ---TVFPNSEIIFCKVHIQRSIEKYFHN |
| | Emoshkovskii_mosh004g04.q1k | -EYLN--------SPDIIICDRSNAQFR-AIQ---TVFPNSEIIFCKVHIQRSIEKYFHN |
| | Einvadens_EMULE_Ei1 | -KFYD---------LTVVICDRCLSQTN-ALL---QVFKDVNLVFCTRHIRRNLITEFGK |
| | Einvadens_inv035b12.q1k | -KKVN---------LTSVYCDRCFSQTK-ALE---TSFNGISIFHCVRHIRRDLQVAFGK |
| ***Hop/Jittery*** | Foxysporum_30421204_Hop78 | -SVYEDH---NIGLPSVILTDRCLACMN-AVS---SCFPGSALFLCLWHINKAVQSYCRP |
| | Ylipolitica_50553866_Mutyl | -VFLQKY---LIPDPGVILSD-CAPAIKKGHKY--SELNGSNLKLCLWHAAKAVERKGS- |
| | Zmays_19908843 | -TCMG------SEGPRVMLTDQDPAMPI-ALR---TVFPKTVHRLCLWHVQNRFMPFLNE |
| | Osativa_50904427 | -TAMG------GKHPETIITDQDLAMRA-AIR---QVFPNSKHRNCLFHILKKCRERSGN |
| | Athaliana_5764395 | -RAMG------GRAPKVILTDQDKFLMS-AVS---ELLPNTRHCFALWHVLEKIPEYFSH |
| | Zmays_7673677_Jittery | -VAMG------GKAPRLIITDEDASIKS-AIR---TTLPDTIHRLCMWHIMEKVSEKVGH |
| | Osativa_50931191 | -KATG------GVAPRLIITDEDASMKA-AIA---QILPNTVHRLCMWHIMEKVPEKVGP |
| | Cglobosum_116196930 | -SLYEVC---NARTPSVVLTDRCIACMN-AVS---TCFPSAASLLCLWHANKAILRHCQP |
| | Mtruncatula_124361025 | -KLLSS----KMNMPKVIVTDRDMSLMK-AVA---HVFPESYALNCFFHVQANVKQRCVL |
| | Cneoformans_58264436 | -ELVG-----KPDV-KVVITDRDPALIN-ALM---SVLPKAYRFSCFWHLQENVKSNIRP |
| | Ptritici_189208826 | -ELFDYL---SVS-PKCFLTDHDRSLKA-GLS---VIFPGIPQRRCIWHIYQNVQTEAVK |
| | Vvinifera_157338603 | -RAMG------GQAPRVILTDQDKALKE-AIA---EVFPESRHCFCLWHILSKIPEKLSC |
| ***MuDR*** | Zmays_23928448_MURAZC | VVGDL--------PLLALCSDACKGLKN-AMN---NVFPHAEKRECFRHLIQNYIKLFGG |
| | Zmays_540581_MudrA | VVGDM--------TLLAICSDAQKGLMH-AVN---EVFPYAERRECFRHLMGNYVKHHAG |
| | Osativa_34914922 | GIDNT--------YPWTIMTNKQKGLIP-AVK---KVFPDTEHRFCVRHLYSNFQEKFKG |
| | Osativa_37700327 | GIVNT--------YPWTIMTDKQKGLIP-AVQ---QLFPDSEHRFCVRHLYQNFQQSFKG |
| | Athaliana_8777291 | IIADS--------NTLTILSDRHESIKV-GVK---KVFPQAHHGACIIHLCRNIQARFKN |
| | Athaliana_15225608 | AIPDD--------PELVFVSDRHNSVYA-SIR---KVYPMSSHAACVVHLKRNIEASFKY |
| | Athaliana_22331509 | IIKDG--------PDLTFVSDRNQSIFK-VVS---LVFNQAHHGACLVHIRRNVKGRYVI |
| | Athaliana_15224361 | IVPDT--------DDLMIVSDRHSSIYK-GVS---VVYPKANHGACIVHLERNISVSYAR |
| | Athaliana_11994228 | VIPDS--------TELVFMSDRNSSLIK-AVA---EVYPSSHHGNCVYHLSQNVRTKVAY |
| | Athaliana_4220448 | IVQDS--------DDLLIVSDRHSSIYK-GLS---VVYPRAHHGACAVHLERNLSTYYGK |
| | Osativa_34910408 | LIGPN--------REVCIISDRHPGILN-SIIHIMPHHLTIHHRWCMRH----------- |
| | **Tvaginalis_TvMULE1** | FIPPS--------RFKNIYSDQGPAIIA-AVR---ESGFSCDHKFCLRHFATKREYINVY |
| ***TvCaMULEs*** | **Calbicans_68466277** | -HSVT---------LKQVMIDCSMPELS-AIK---TVFPESSVSICKWYILRNVRTEARS |
| | **Calbicans_68466572** | -HSVT---------PKQVMIDCSMSELS-AIK---TVFPESSVSICKWHILRNVRTEARS |
| | **Calbicans_68474652** | -TEFG-------INPTQFVIDADPAEIS-GIQ---SIFKDTKIVLCYFHVLRAVTIKLKE |
| | **Tvaginalis_TvMULE2** | -AHLD---------PQHWSADCALNIAR-AIE---DGFPLAQLSWCAVHVLRACAKVGSY |
| | **Tvaginalis_TvMULE3** | -AKVK---------PKYFMSDCAQEIEN-AII---NSFPEVILHWCAVHVMRAFRKNLKD |
| | **Tvaginalis_TvMULE4** | -INRE---------PSYFSMDCAPQITN-AVE---TAIPLCOIIWCGVHVLRAVMRKAEK |

**Motif 1**
